# Partner testing, linkage to care, and HIV-free survival in a program to prevent parent-to-child transmission of HIV in the Highlands of Papua New Guinea

**DOI:** 10.3402/gha.v7.24995

**Published:** 2014-08-27

**Authors:** Andy Carmone, Korai Bomai, Wayaki Bongi, Tarua Dale Frank, Huleve Dalepa, Betty Loifa, Mobumo Kiromat, Sarthak Das, Molly F. Franke

**Affiliations:** 1 Clinton Health Access Initiative, Goroka, Papua New Guinea; 2 Goroka Family Clinic, Eastern Highlands Provincial Hospital, Goroka, Papua New Guinea; 3 Department of Global Health and Social Medicine, Harvard Medical School, Boston, MA, USA

**Keywords:** rural, vertical transmission, case management, mother-to-child transmission, retention, PMTCT

## Abstract

**Background:**

To eliminate new pediatric HIV infections, interventions that facilitate adherence, including those that minimize stigma, enhance social support, and mitigate the influence of poverty, will likely be required in addition to combination antiretroviral therapy (ART). We examined the relationship between partner testing and infant outcome in a prevention of parent-to-child transmission of HIV program, which included a family-centered case management approach and a supportive environment for partner disclosure and testing.

**Design:**

We analyzed routinely collected data for women and infants who enrolled in the parent-to-child transmission of HIV program at Goroka Family Clinic, Eastern Highlands Provincial Hospital, Papua New Guinea, from 2007 through 2011.

**Results:**

Two hundred and sixty five women were included for analysis. Of these, 226 (85%) had a partner, 127 (56%) of whom had a documented HIV test. Of the 102 HIV-infected partners, 81 (79%) had been linked to care. In adjusted analyses, we found a significantly higher risk of infant death, infant HIV infection, or loss to follow-up among mother–infant pairs in which the mother reported having no partner or a partner who was not tested or had an unknown testing status. In a second multivariable analysis, infants born to women with more time on ART or who enrolled in the program in later years experienced greater HIV-free survival.

**Conclusions:**

In a program with a patient-oriented and family-centered approach to prevent vertical HIV transmission, the majority of women's partners had a documented HIV test and, if positive, linkage to care. Having a tested partner was associated with program retention and HIV-free survival for infants. Programs aiming to facilitate diagnosis disclosure, partner testing, and linkage to care may contribute importantly to the elimination of pediatric HIV.

As part of the Global Plan Toward Elimination of New HIV Infections Among Children By 2015 and Keeping Mothers Alive, UNAIDS set a goal of reducing new HIV infections among children by 90% between 2011 and 2015 (1). Chief among the requisites for achieving this target are widely accessible, effective programs to provide antiretroviral therapy (ART) to HIV-infected pregnant women early in pregnancy, and to those desiring children (1). While the effectiveness of ART for the prevention of mother-to-child transmission of HIV (PMTCT) is clear, many programs report high rates of loss to follow-up (2, 3) and struggle to achieve the ≤2% transmission rates that have been shown to be attainable (4–10). Highly variable PMTCT success rates across programs likely represent the complex, multifaceted factors that contribute to access to care and early initiation of and adherence to ART (11–14). They also suggest that, in order to further drive down transmission rates, some PMTCT programs may need to incorporate interventions aimed at curtailing the effects of stigma, enhancing social support, and reducing the influence of poverty.

Involvement of male partners in PMTCT is one promising strategy to further reduce HIV vertical transmission and improve infant survival (15, 16). Greater uptake of PMTCT interventions and adherence to the feeding option of choice has been demonstrated with male partner involvement 17–22). Nonetheless, the HIV disclosure process may be complex, and some women may feel unequipped to disclose in the absence of additional support (23). By disclosing her HIV status to her partner, a woman may put herself at risk for violence (23–25); abandonment – this fear may be especially acute for women in polygamous relationships (23, 24, 26); and loss of reputation (27) or material or economic support (23, 24, 26). In Tanzania, reliance on a partner for expenditures such as food, rent, or school fees was associated with lower rates of HIV diagnosis disclosure (28). Once disclosure does occur, barriers for HIV testing among male partners include the perception of PMTCT clinics as a woman's place (29, 30); the time required for testing, which may interfere with employment (31); and fear that he will be blamed for introducing HIV into the family.

In 2006, the Papua New Guinea (PNG) Department of Health and Clinton Health Access Initiative developed a rural service delivery model to provide HIV care and treatment in rural PNG. Now termed the Patient and Provider Unified Approach (PAPUA), the model seeks to address important patient barriers to health, including difficult access to health care, HIV-related stigma, and extreme poverty, while also emphasizing the need for stronger provider support through training, mentorship, and supervision (32). PAPUA includes a comprehensive, case management–led PMTCT program, which provides support in diagnosis disclosure to partners and outreach to partners and children for testing and support. In 2007, the government of PNG replaced the conventional nomenclature “Prevention of Mother-to-Child Transmission of HIV” with “Prevention of Parent-to-Child Transmission of HIV” (PPTCT). At that time, national antenatal HIV-testing rates were low (33), and there was a fear among health workers and leaders that pregnant women diagnosed with HIV could be blamed or victimized by their male partners or families and face physical violence or indirectly violent exclusionary behavior (34). Replacing the word “mother” with “parent” was intended to both acknowledge the role of the male partner in the process of prevention of vertical transmission and lighten any potential stigma for women. To our knowledge, there are no published papers examining PPTCT outcomes in PNG, let alone in the rural areas, where the majority of HIV-infected persons in PNG reside (35). The objectives of this study were to quantify the uptake of HIV testing and care among partners of HIV-infected pregnant women receiving care in this program, to examine the relationship between partner testing status and program retention with infant HIV-free survival, and to identify other factors associated with HIV-free survival.

## Methods

### Study setting

PNG, an island of 7 million inhabitants, has the highest burden of HIV in the South Pacific (36). In the country's rural Highlands areas, PPTCT is particularly challenging due to high rates of HIV-related stigma, gender inequality, and poverty (37). Polygamy, concurrent partnerships, and intimate partner violence are common in these areas (34, 38, 39). This study was conducted in the Eastern Highlands Province, where the life expectancy is 55 years, 44% of adults are literate, and only 45% of the population lives within 5 km of a national roadway (40).

### Study population

Included in this analysis were all women who were registered for PPTCT services at any time during pregnancy, including during labor, from 2007 through 2011 at Goroka Family Clinic at the Eastern Highlands Provincial Hospital, PNG. We excluded women for any of the following reasons: 1) her chart could not be located; 2) her infant's chart could not be located, and infant status could not be determined; or 3) she was found not to be pregnant or not to be HIV infected. Women and infants who presented to the clinic for the first time after delivery were not enrolled in the PPTCT program, but rather treated through the pediatric clinic. Therefore, these women and their infants were not included in this study.

### PPTCT standard of care throughout the study period

The PNG National Guidelines for HIV Care and Treatment were used to direct clinical care and ART for pregnant women during the study period (41). The recommended first-line triple-therapy regimen for HIV-infected pregnant women requiring ART for their own health or PPTCT was zidovudine, lamivudine, and nevirapine. Guidelines for PPTCT during the study period were as follows. Prior to 2009, guidelines indicated that women who did not require ART for their own health be commenced on daily single-dose zidovudine, followed by intrapartum and “tail” doses of lamivudine and nevirapine. After 2009, women were recommended to initiate triple therapy after 28 weeks of gestation. Women who did not initiate triple therapy during pregnancy were given a single dose of nevirapine plus lamivudine and zidovudine at the onset of labor, followed by a single dose of nevirapine to the infant. Four to six weeks of zidovudine was prescribed to all infants. Throughout the study period, it was recommended that women receiving triple therapy for PPTCT continue it for the duration of breastfeeding to prevent postnatal transmission. HIV testing was recommended for women attending antenatal clinic throughout the study period, with opt-out testing beginning in 2007. Partner testing was recommended for women enrolled in PPTCT services beginning in 2009.

### Partner testing and case management in PAPUA

This PPTCT program was part of a provincial network of centrally-coordinated decentralized HIV service delivery centers (32). Antenatal testing for HIV, syphilis, and hemoglobin was provided by linked clinics serving the populace of Eastern Highlands in 20 locations, 10 of which also provided adult ART. HIV-infected pregnant women were referred to a central antenatal–PPTCT–pediatric clinic, located at the provincial hospital in the capital city.

As part of PAPUA, PPTCT services were structured around treatment of the HIV-infected woman and all of her family members, unless the woman chose not to disclose her diagnosis. Focus on the family, particularly male partners, within the mother's care was appropriate for the local Highlands cultures, where men usually make decisions, yet women provide most of the supporting work (42). The addition of a case management system to the usual clinical care aimed to assure linkages; provide ongoing, in-depth patient counseling; remove material obstacles to care; and ensure a higher quality care. Case managers aimed to build a trusting environment where women could feel supported and safe enough to consider disclosure of their HIV status to male partners and possibly to other family members. After initial relationship building, case managers encouraged women to bring in their male partners and children for counseling and testing. Case managers would sometimes test women and their male partners together as if the woman had not yet been tested. Some women selected this approach because it allowed for both partners’ results to be reported at the same time and with the presence and support of the case manager, which could minimize accusations and blame. Synchronization and consolidation of clinical appointments with regular antenatal services was a cornerstone of case management and allowed for appointments for antenatal and HIV care to occur on the same day and in the same place for women, HIV-infected male partners, and HIV-exposed infants. This approach was especially valuable to families traveling from farther districts within the province, as it required only one monthly visit per family. Women and families received reimbursement for transportation costs, as well as modest nutritional support, and family-sized mosquito nets.

### Data collection

We conducted a retrospective review of existing programmatic and clinical records. The PPTCT registration book was used to identify women eligible for inclusion in these analyses. Data from mothers’, infants’, and partners’ paper charts were reviewed and transcribed to standardized study forms, using electronic data and clinician interviews to fill in gaps as needed.

### Definitions

Partner status was classified into three categories: no partner, tested partner, and partner who was untested or had an unknown testing status. Program completion was determined as follows: for replacement-fed infants, an HIV-negative HIV DNA polymerase chain reaction (PCR) test after 6 weeks of age was required; for breastfed infants, an HIV-negative test 6 weeks after the wean date was required, and, if no wean date was available, either two negative tests or one negative test after 15 months of age was required. Infants who died, mothers who became lost to follow-up during pregnancy, and mother–infant pairs who were lost after delivery were not considered to have successfully completed the program. Partners were determined to have been linked to care if they could be found in the HIV registry or had a chart in an adult HIV clinic. In the absence of an ART start date indicating the contrary, women who were on ART for their own health previously were assumed to have been on treatment for the duration of their pregnancy.

### Statistical analysis

We conducted regression analyses to examine the relationship between partner testing status and the study outcome: program retention with HIV-free survival and its inverse (i.e. lost to follow-up, infant death, or infant HIV infection). We estimated risk ratios using the simple strategy proposed by Tchetgen Tchetgen (43). Women who experienced a stillbirth, mother–infant pairs who transferred out of the program, and mother–infant pairs for whom we lacked an infant chart or partner data were excluded from multivariable analyses. Because having a tested partner may influence a woman's decisions about a number of issues on the causal pathway to infant health outcomes (i.e., earlier presentation to the clinic, longer time on ART at delivery, more antenatal visits, and better adherence to treatment), we adjusted only for maternal age, time with HIV diagnosis at the first antenatal visit (greater than or less than 1 year), and year of program enrollment. Missing covariate data were multiply imputed using Markov chain Monte Carlo methods to complete the data set. We used the same procedures to conduct a secondary analysis of other factors associated with infant HIV-free survival. For this analysis, we additionally excluded women who became lost to follow-up, either prior to or after delivery. Any variable associated with HIV-free survival at a *p*<0.20 in univariable analyses was included in the multivariable analyses. When variables were correlated, we retained that most strongly associated with the outcome. Analyses were conducted using SAS version 9.12 (SAS Institute, Cary, NC, USA).

## Results

**Fig. 1:**
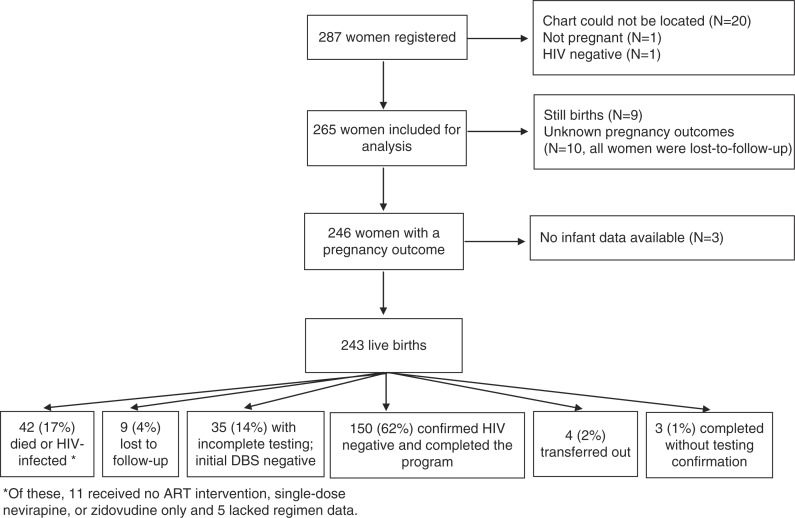
Overview of PPTCT registration and outcomes, Eastern Highlands Province, Papua New Guinea, 2007–2011.

Two-hundred eighty-seven women were registered for PPTCT services during the study period. Of these, charts could not be located for 20. Additionally, one woman was determined not to be pregnant, and one was determined to be HIV-negative. An overview of the mother–infant pairs comprising the study cohort is shown in Figure 1.

Characteristics of the 265 women included for analysis are shown in Table 1. The median age was 24 years (interquartile range [IQR]: 21–29). Women tended to present to the PPTCT clinic late in gestation with a median gestational age of more than 6 months at the time of the first visit (median: 197.5 days; IQR: 153.5–232.5). Women received ART for a median of 83 days prior to delivery (IQR: 22–274), and 12.0% of women received only single-dose nevirapine or did not receive an ART intervention. Gestational age at first antenatal visit and time on ART before delivery were inversely correlated (correlation=−0.43, *p*<0.0001). The number of women registered for PPTCT was greater in later years.

**Table 1:** Characteristics of 265 women registered for prevention of parent-to-child HIV transmission services in Goroka, Papua New Guinea

Variable	*N* with data	*N* (%) or median (IQR)
Age (years)	259	24 (21–29)
Time from diagnosis to first antenatal visit (days)	224	53 (8.5–509)
Gestational age at first antenatal visit (days)	200	197.5 (153.5–232.5)
Number of antenatal visits	265	4 (2–7)
Initiated ART for own health	250	75 (30.0)
Antiretroviral regimen	250	
ZDV, 3TC, NVP		163 (65.2)
D4T, 3TC, NVP		39 (15.6)
ZDV, 3TC, EFV		2 (0.8)
Triple therapy, unspecified		3 (1.2)
ZDV only		13 (5.2)
Single dose NVP		19 (7.6)
No ART^a^		11 (4.4)
Time on ART prior to delivery (days)	211	83 (22–274)
Ever had a suboptimal adherence assessment (<95% adherence)	247	18 (7.3)
CD4 count during pregnancy	132	276 (217.5–380.5)
Lymphocyte count during pregnancy	152	1300 (1000–1900)
HIV stage before delivery	160	
1		127 (79.4)
2		18 (11.3)
3		13 (8.1)
4		2 (1.3)
Year of first antenatal visit	246	
2007		19 (7.7)
2008		40 (16.3)
2009		62 (25.2)
2010		72 (29.3)
2011		53 (21.5)
Feeding method^b^	196	
Exclusive breastfeeding		136 (69.4)
Formula feeding		36 (18.4)
Mixed feeding		24 (12.2)

a Some women who had no record of ART prior to delivery may have received ART during delivery without it being recorded in the chart.

b Among women who had a live birth. IQR=interquartile range; ART=antiretroviral therapy; ZDV=zidovudine; EFV=efavirenz; NVP=nevirapine.

### HIV testing uptake and linkage to care

**Fig. 2:**
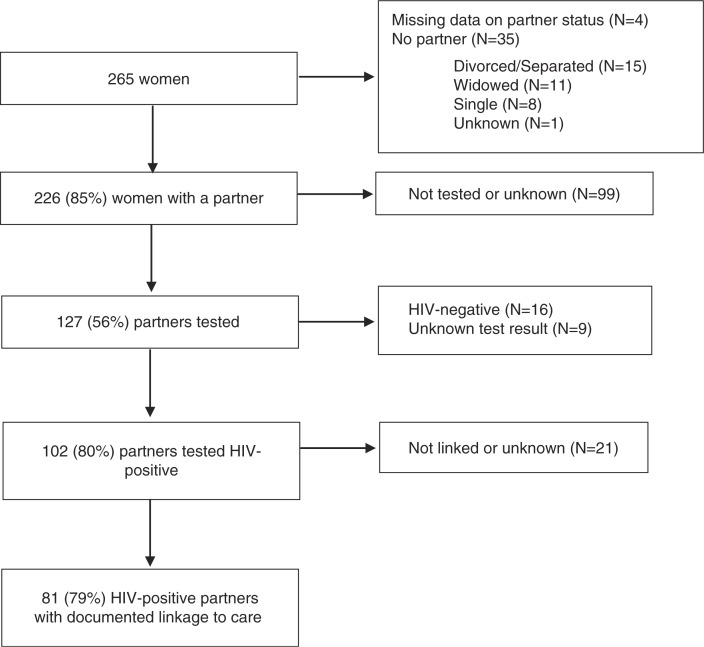
Linkage to care among partners of women registered for PPTCT services, Eastern Highlands Province, Papua New Guinea, 2007–2011.

Figure 2 shows the rate of uptake of HIV testing among partners and their linkage to care. Among the 265 women, partner data were available for 261 (98%). Of these, 35 (13%) reported no partner. Of the 226 women with a partner, 127 partners (56%) had an HIV test result, 102 (80%) of which were positive. The vast majority of HIV-infected partners (79%) had a documented linkage to care. On average, women were 5 years younger than their HIV-infected partners (range −5–36). Reasons for no HIV testing among partners were not generally available but were noted for 22 women and included living apart from the partner (*n*=13), not wanting to disclose to the partner (*n*=8), and having a partner who did not want to be tested (*n*=1).

### Partner testing and program retention with HIV-free survival

Nine women whose infants were stillborn, four who transferred out of the program prior to completion, three with infants for whom we lacked a chart, and four women lacking data on partner information were excluded from multivariable analyses. Among 245 mother–infant pairs, 150 women successfully completed the program with an infant who was alive and documented as HIV-free. Partner status (i.e. no partner, tested partner, partner not tested, or partner with an unknown status) was not associated with maternal age (Kruskal Wallis *p*=0.20), but was associated with time since HIV diagnosis. Individuals with a tested partner were more likely to have had their HIV diagnosis for at least a year, whereas individuals with an untested partner or a partner of unknown HIV status were more likely to have been diagnosed in the 12 months prior to PPTCT enrollment (chi-squared *p*=0.001). Not having a partner or having a partner who was not tested or had an unknown testing status was associated with an increased risk of loss to follow-up, infant death, or infant HIV infection in both univariable analysis and a multivariable analysis adjusting for maternal age, time with HIV diagnosis at first antenatal visit, and year of program enrollment (Table 2).

**Table 2:** Partner-testing status and outcome of loss to follow-up, infant death, or infant HIV infection (*N*=245)^a^

	*N*	Outcome of loss to follow-up, infant death, or infant HIV infection *N* (%)	Univariable OR (95% CI)	*p*	Multivariable OR (95% CI)^b^	*p*
Has tested partner	118	35 (29.7)	Reference	–	Reference	
No partner	30	15 (50.0)	1.69 (1.07, 2.65)	0.02	1.59 (1.01, 2.51)	0.05
Has partner, not tested or unknown	97	47 (48.5)	1.63 (1.16, 2.31)	0.005	1.50 (1.05, 2.16)	0.03

a Women with a stillbirth (*N*=9), who were transferred out of the program prior to completion (*N*=4), or for whom we lack an infant chart (*N*=3) or partner data (*N*=4) were excluded from analysis.

b Adjusted for maternal age, time with HIV diagnosis at first antenatal visit (greater or less than 1 year), and year of enrollment.

### Factors associated with HIV-free survival

Table 3 shows factors associated with HIV-free survival in univariable and multivariable analyses. Among the 230 women included, 42 gave birth to infants who later died or were confirmed to be HIV infected (Fig. 1). In univariable analysis, longer time on ART, later year of program enrollment, having initiated ART for her own health, and a greater number of antenatal visits were all positively associated with infant HIV-free survival, whereas older gestational age at first antenatal visit was negatively associated with HIV-free survival. In multivariable analysis, longer time on ART and later year of program enrollment were positively associated with HIV-free survival. Excluding mother–infant pairs who were lost to follow-up, infants born to the 67 women who had more than 24 weeks of ART had an HIV-free survival percentage of 96%. In contrast, the 45 infants born to women with an unknown ART duration and the 34 infants born to women with less than 1 week of ART had HIV-free survival percentages of 60 and 62%, respectively.

**Table 3:** Predictors of HIV-free survival (*N*=230^a^)

	Univariable RR (95% CI)	*p*	Multivariable RR (95% CI)	*p*
Time on ART prior to delivery (weeks)	1.006 (1.003, 1.009)	0.0004	1.01 (1.00, 1.01)	0.005
Age (5-year increase)	1.06 (0.99, 1.13)	0.11	1.03 (0.97, 1.11)	0.35
Year of first antenatal visit	1.08 (1.03, 1.14)	0.004	1.06 (1.00, 1.13)	0.05
Gestational age at first antenatal visit (months)^b^	0.97 (0.94, 1.00)	0.05		
Number of antenatal visits	1.03 (1.01, 1.05)	0.001	1.02 (1.00, 1.04)	0.06
Ever had a suboptimal adherence assessment	0.95 (0.73, 1.24)	0.71		
Feeding method				
Mixed feeding	Reference			
Exclusive breastfeeding	0.99 (0.87, 1.13)	0.89		
Formula feeding	0.97 (0.82, 1.15)	0.69		
Initiated ART for her own health^b^	1.22 (1.11, 1.35)	<0.0001		

a Women with a stillbirth (*N*=9), who were transferred out of the program prior to completion (*N*=4), who were lost to follow-up (*N*=19), or for whom we lacked an infant chart (*N*=3) were excluded from analysis. The sample size for the univariable risk ratios changed depending on the number of women missing data for the predictor. The sample size was 230 for the multivariable analysis, which was conducted on multiply imputed datasets.

b Gestational age at first antenatal visit and initiation of ART for the woman's own health were correlated with time on ART prior to delivery and therefore not included in the final multivariable model.

## Discussion

In the context of a PPTCT program that included a case management intervention to facilitate diagnosis disclosure to partners and encourage partner testing, a high percentage of women's partners were tested (56%) and tested positive for HIV (80%). Among those partners with an HIV diagnosis, the majority had linkage to care. Furthermore, mother–infant pairs were more likely to complete the program with infant HIV-free survival if the mother had a tested partner. By demonstrating a positive relationship between partner testing and infant health outcomes, this study expands upon prior work indicating that uptake of and adherence to PPTCT interventions may be jeopardized by nondisclosure to male partners or lack of male partner involvement (19–22). Consistent with our findings, Aluisio and colleagues found that infants born to HIV-infected women whose male partners attended antenatal clinic visits experienced improved survival relative to infants born to HIV-infected women whose partners did not attend antenatal clinic visits (15).

The success and uptake of couples HIV testing may vary by setting and implementation strategy. Couples counseling and testing and facilitated mutual disclosure, in either the home or clinic setting, were found to be acceptable in Kenya (44); however, a randomized trial from Tanzania found that couples HIV testing, relative to individual testing, led to fewer women returning for test results (45). Additional strategies to facilitate male partner participation in PPTCT services could include clinic hours and physical spaces that are accommodating and appealing to men, sensitization of both health care providers and men to the importance of male partner involvement in PPTCT, targeted messaging toward those men least likely to participate in PPTCT, and interventions, such as screening for domestic violence and mental health problems, which may contribute to enhanced family functioning (16, 30, 46, 47).

Women who did not have a partner and those whose partner was not tested were the majority of the women included in this analysis, and they represent key groups that may need extra support in order to successfully complete PPTCT programs. Pregnant women without a partner may be widows, be victims of rape, or have had transactional sex outside of a formal relationship. All three of these scenarios indicate a high level of vulnerability that may challenge the ability to access health services and adhere to treatment. Though we lacked systematically collected data on partner disclosure, it is possible that male partners who were not tested or had unknown testing status did not have knowledge of their partners’ diagnoses. Furthermore, each woman must consider the benefits of disclosure and partner testing against the possible negative consequences, which include suspected infidelity, loss of sexual intimacy, conflict, stigma, violence, or loss of community standing (24, 48).

Late presentation to the PPTCT program led to suboptimal exposure to ART prior to delivery for some women and posed a major challenge to PPTCT in this setting. Longer time on ART prior to delivery was strongly associated with better HIV-free survival. Among those who had more than 24 weeks of ART prior to delivery, 96% of infants were alive and HIV-free when they exited the program. Program maturation, changing treatment guidelines, and greater confidence and experience among providers may have contributed to improved HIV-free survival over time. In addition to initiation of ART early during pregnancy, ensuring an adequate drug supply was another challenge that interfered with optimal ART exposure for pregnant women. Three significant ART stockouts occurred during the study period, and it likely prevented timely initiation of some onto treatment and interrupted therapy in some who had already commenced treatment.

Beginning in 2007, a decentralized basic package of antenatal testing for HIV, syphilis, and hemoglobin from the referral hospital to 20 satellite district clinics was rolled out throughout the study province. While this step was critical to improving case finding for women in need of PPTCT, additional services including ART, HIV-appropriate delivery services, and partner testing were not available at these district sites during the study period. With programs reporting better uptake when ART was provided at the same place as PPTCT services (49, 50), we expect that the decentralization of the full complement of PPTCT services will further improve HIV-free survival for infants in the program.

Linking records for mothers, infants, and partners sometimes involved tracking paper records across multiple clinics and sites. Though largely successful, not all charts could be located. Imperfect tracing and the tendency for adults in this setting to change their names to ensure anonymity in HIV clinics likely resulted in underestimates of true linkage-to-care rates. A second important limitation of this analysis is that women with tested partners may differ from women without tested partners in ways that also influence program retention and adherence to medication, such as familial support and livelihood security. We lacked data on socioeconomic indicators, intrafamilial violence, and family structure and therefore cannot discount the possibility that unmeasured differences between women with and without tested partners explain the associations observed here. A third limitation is that, due to a large amount of missing data among the partner-testing data, we could not confirm which partners were tested as part of the PPTCT case management partner-testing program versus through another mechanism, when partner HIV tests occurred relative to the pregnancy, and when partners’ linkages to care occurred relative to the pregnancy. It is possible that some HIV-infected men encouraged their female partners to seek HIV testing and treatment before or during pregnancy. Finally, the retrospective nature of this analysis led to substantial missing data for some variables, particularly dates. In spite of these limitations, the results presented here point to the importance of partner engagement and the potential role of robust partner-testing programs in the elimination of perinatal transmission of HIV.

## Conclusions

We found relatively high rates of partner testing among HIV-infected women in this setting. Pregnant women with a tested partner were more likely to have a child who completed the program and remained alive and HIV-negative. When the primary purpose of HIV care and treatment is prevention of vertical transmission of HIV, with the secondary purpose of providing a gateway to care for parents, patient-oriented and family-centered comprehensive service delivery contributes to HIV-free infant survival, PPTCT program completion, and linkage of positive male partners into care. From the perspective that healthy and well-supported parents, mothers in particular, are the building blocks for healthy children, PAPUA's approach to PPTCT may have important lessons for other such programs.
